# High mast cell density indicates a longer overall survival in oral squamous cell carcinoma

**DOI:** 10.1038/s41598-017-15406-5

**Published:** 2017-11-07

**Authors:** Phillipp Brockmeyer, Alexander Kling, Xenia Schulz, Christina Perske, Henning Schliephake, Bernhard Hemmerlein

**Affiliations:** 1 Department of Oral and Maxillofacial Surgery, University Medical Centre Goettingen, Goettingen, Germany; 2 Department of Medical Statistics, University Medical Centre Goettingen, Goettingen, Germany; 3 Department of Pathology, University Medical Centre Goettingen, Goettingen, Germany; 4 Institute of Pathology, Helios Klinikum Krefeld, Krefeld, Germany

## Abstract

This study evaluates the effects of tumour-associated mast cells on the prognosis of patients suffering from oral squamous cell carcinoma (OSCC). Tryptase-positive (MCT^+^) and CD117-positive (CD117^+^) mast cells were immunohistochemically evaluated in tissue samples of 118 OSCC patients. Besides, various clinicopathological parameters, the influence of the MCT^+^ and CD117^+^ mast cell density on overall survival and the incidence of first local recurrence was analysed by Cox regression and competing risk regression. Among all investigated parameters, multiple Cox regression revealed a significant influence of the MCT^+^ (cut-off at 14.87 mast cells/mm^2^ stroma; p = 0.0027) and CD117^+^ mast cell density (cut-off at 33.19 mast cells/mm^2^ stroma; p = 0.004), the age at primary diagnosis, and the T and N stage (all p-values < 0.05) on overall survival. Patients with a low mast cell density showed a significantly poorer overall survival rate compared to those with a high mast cell density in the tumour-associated stroma. Competing risk regression revealed a significant influence of the resection status (R) on the incidence of first local recurrence (p = 0.0023). A high mast cell density in the tumour-associated stroma of oral squamous cell carcinoma indicates a longer patient survival.

## Introduction

Besides their important function as potent effector cells of the immune system^1^, mast cells can support as well as suppress tumour development and progression^2^. A poor prognosis has been associated with increased mast cell density in patients suffering from Hodgkin’s lymphoma^3,4^, malignant melanoma^5,6^, and various types of carcinomas including squamous cell carcinoma of the esophagus^7^, lung adenocarcinoma^8^, and gastrointestinal adenocarcinoma^9,10^. Mast cells can release angiogenic factors (e.g. vascular endothelial growth factor) from their granules in the tumour stroma, supporting early angiogenesis, while the release of histamine can induce tumour cell proliferation^11,12^. Moreover, mast cells release matrix metalloproteinases and proteases like tryptase and chymase which degrade the extracellular matrix and promote tumour spread and metastasis^11–13^. Mast cells can directly and indirectly suppress the immune system, which promotes tumour spread via the release of IL-10 and TGF-β1^13^. In contrast, a high intratumoural mast cell density has been described to be associated with a favourable prognosis in prostate^14,15^, colorectal^16,17^, and clear-cell renal cell carcinoma^18^. Mast cells have a TNF-induced cytotoxic effect on tumour cells^13^ and promote apoptosis^12^; via the release of different interleukins such as CCL5, CXCL8, CXCL10, and IL-6, they can recruit and activate various immune cells that inhibit tumour growth^13^. These different findings strongly depend on mast cell localization and whether they are in close contact with tumour cells or located in the tumour stroma^2,19,20^. To evaluate the effects of tumour-associated mast cells on the prognosis of patients suffering from OSCC, we analysed the relevance of the mast cell density in the tissue samples of 118 patients compared to a comprehensive spectrum of clinicopathological parameters by using a multivariable statistical approach.

## Materials and Methods

### Patients

OSCC tissue samples of 118 patients who had primarily been treated surgically during 1995–2007 were used for immunohistochemical evaluation. The patients gave written informed consent before participating in the trial. The study was conducted in accordance with the ethical standards (Declaration of Helsinki) approved by the local ethics committee of the University of Goettingen (vote number 07/06/09). The patient characteristics used for evaluation are summarized in Table 1.

**Table 1:** Clinicopathological parameters. Absolute and relative frequency of categorical variables and mean +/− standard deviation; median (minimum, maximum) of metrical variables.

Parameter	Level	Descriptive Statistics
Alcohol abuse	Yes	69 (62.7%)
	No	41 (37.3%)
Age at primary diagnosis (years)		60.81+/− 11.98; 62 (31,94)
Body Mass Index (BMI)		23.83+/− 4.37; 23 (14,41)
CD117^+^ mast cells/mm^2^ stroma		34.46+/− 29.95; 27.85 (0.63,208.29)
Histopathological grading	G1	17 (14%)
	G2	95 (78.5%)
	G3	9 (7.4%)
Body weight (kg)		69.36+/− 14.02; 70 (36,104)
Body height (m)		1.7+/− 0.08; 1.72 (1.51,1.98)
Localisation of primary OSCC	Alveolar process/jaw	36 (30%)
	Palate/oropharynx	6 (5%)
	Mouth floor	44 (36.7%)
	Cheek/lip	9 (7.5%)
	Tongue	25 (20.8%)
MCT^+^ mast cells/mm^2^ stroma		38.24+/− 32.59; 31.56 (0.23,238.3)
N stage	Negative	87 (71.9%)
	Positive	34 (28.1%)
Lymphadenectomy (left and/or right side)	Performed	147 (62.0%)
	Not performed	90 (38.0%)
Nicotine abuse	Yes	87 (76.3%)
	No	27 (23.7%)
R status	R0	113 (93.4%)
	R1	8 (6.6%)
Reconstruction modality	Distant flap	53 (46.1%)
	Local	62 (53.9%)
Side of primary OSCC	Left & right	101 (84.2%)
	Median	19 (15.8%)
Sex	Male	82 (67.8%)
	Female	39 (32.2%)
AJCC stage	1 & 2	45 (37.2%)
	3 & 4	76 (62.8%)
T stage	1 & 2	57 (47.1%)
	3 & 4	64 (52.9%)
Deceased	No	54 (44.6%)
	Yes	67 (55.4%)

### Tissue samples

**Figure 1:**
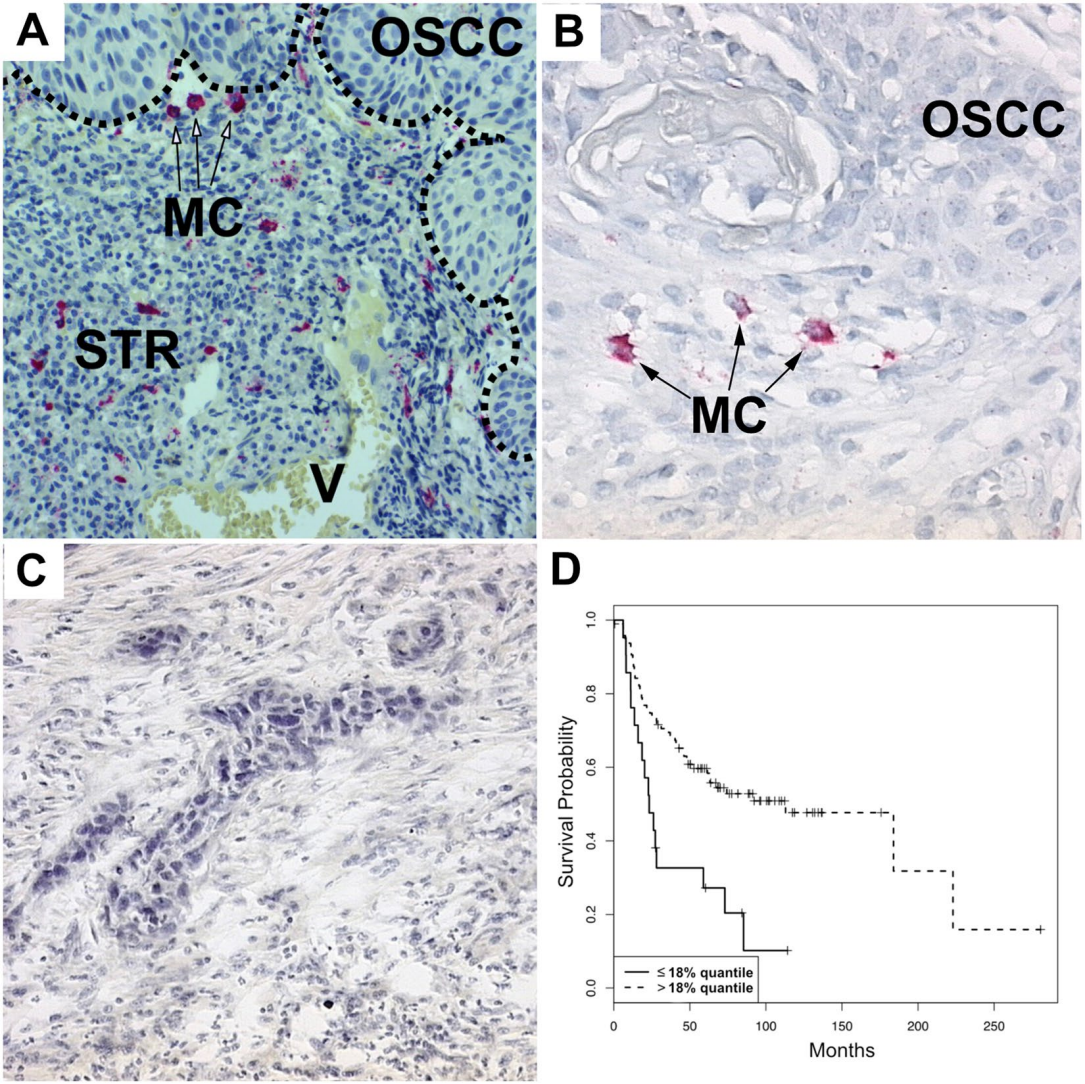
(**A**) Illustration of MCT immunohistochemistry (magnification factor × 200), border between OSCC and stroma (STR) (dotted line), vessel with erythrocytes (V), mast cells (MC); (**B**) Illustration of MCT immunohistochemistry (magnification factor × 400); (**C**) Mouse IgG2b monoclonal isotype control (magnification factor × 400); (**D**) Probability of overall survival stratified by MCT mast cell density, breakdown of distribution according to the 18% quantile.

**Figure 2:**
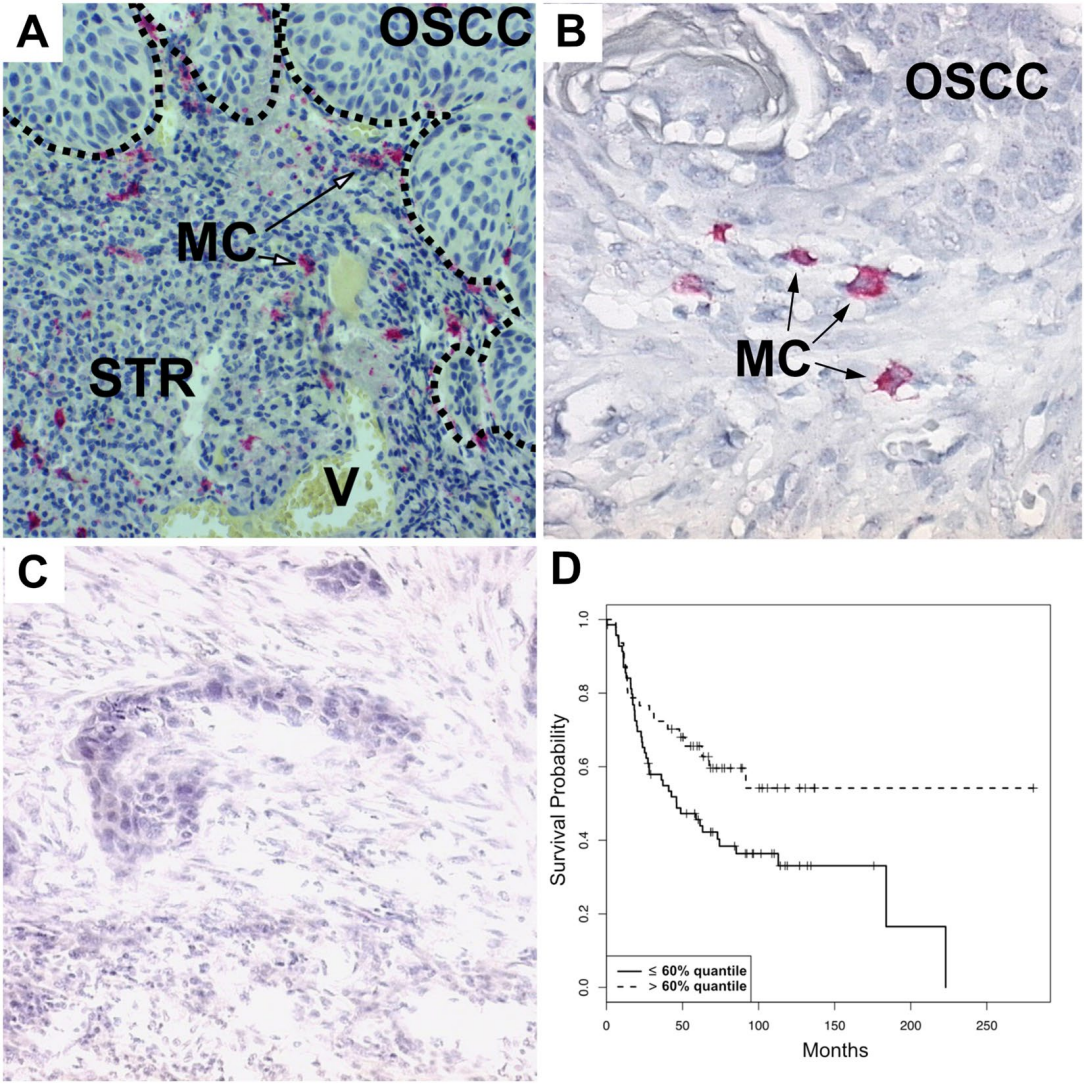
(**A**) Illustration of CD117^+^ immunohistochemistry (magnification factor × 200), border between OSCC and STR (dotted line), vessel with erythrocytes (V), mast cells (MC); (**B**) Illustration of CD117^+^ immunohistochemistry (magnification factor × 400); (**C**) Rabbit IgG monoclonal isotype control (magnification factor × 400); (**D**) Probability of overall survival stratified by CD117^+^ mast cell density, breakdown of distribution according to the 60% quantile.

Tissue samples obtained during tumour resection were fixed in neutrally buffered 4% formalin and embedded in paraffin. MCT^+^ and CD117^+^ mast cells were immunohistochemically stained on 2 μm tissue sections using an automated slide staining system (BOND-III, Leica, Nussloch, Germany). To exclude non-specific staining, additional isotype control staining was carried out in 20 of the 118 cases as seen in Figs 1C and 2C. We used antibodies which are certificated for *in vitro* diagnosis (Table 2). Representative regions of interest of the tumour-associated stroma were determined in each preparation by a blinded investigator A. The tumour-to-stroma ratio was determined in 10x magnification by using a point-sampling optical grid (Olympus, Tokyo, Japan) in each region of interest. The total area of the region of interest was calculated separately for the MCT^+^ and CD117^+^ sections by using the known area of the optical field. MCT^+^ and CD117^+^ mast cells were counted at 400x magnification in each region of interest in all tissue samples by a second blinded investigator B by using an Olympus BX41 light microscope (Olympus, Tokyo, Japan). Data were controlled regarding its plausibility and validity in a random sample survey by investigator A, respectively. Only signals from mast cells with visible cell nuclei were considered and only complete optical fields were counted. Counted MCT^+^ and CD117^+^ mast cells were reported per mm² stroma. The mean mast cell numbers from all individual regions of interest of the tissue section were used for statistical evaluation. Because mast cells were mainly found in the tumour stroma, and only a very small proportion in the intratumoural cell clusters, we determined the relative amount of stroma within the region of interest by using the following equation:

$${\rm{Mast}}\,{\rm{cell}}\,{{\rm{number}}}_{{\rm{stroma}}}={\rm{total}}\,{\rm{mast}}\,{\rm{cell}}\,{{\rm{number}}}_{{\rm{region}}{\rm{of}}{\rm{interest}}}/({\rm{total}}\,{{\rm{area}}}_{{\rm{region}}{\rm{of}}{\rm{interest}}}\times {{\rm{ratio}}}_{{\rm{stroma}}}).\phantom{\rule{4em}{0ex}}$$

**Table 2:** Used antibodies.

Antigen	Antibody (Type, dilution)	Detection method	Source
Anti-mast cell tryptase, clone 10D11	mouse, monoclonal, 0.03 mg/l, ready-to-use dilution	Bond Polymer Refine Red Detection	Leica, Nussloch, Germany
	Mouse IgG2b monoclonal isotype control, 0.03 mg/l	Bond Polymer Refine Red Detection	Dianova, Hamburg, Germany
Anti-CD117, clone EP10	Rabbit, monoclonal, 0.13 mg/l, ready-to-use dilution	Bond Polymer Refine Red Detection	Leica, Nussloch, Germany
	Rabbit IgG monoclonal isotype control, 0.13 mg/l	Bond Polymer Refine Red Detection	Abcam, Cambridge, United Kingdom

Example calculation:

*Total area*_*region of interest*_ = *10 mm²*, *ratio*_*stroma*_ *60*% = *0.6; Total number of CD117* ^+^ *mast cells in region of interest* = *150*. ***Mast cell number***_***stroma***_ = ***150***/(***10*** **×** ***0*** *.* ***6***) = ***25***/***mm²***

### Statistical analysis

A simple Cox regression was performed for different MCT^+^ and CD117^+^ cut-off values to find the quantile resulting in a significant influence on the overall survival and a low hazard ratio. These binary variables were used, instead of the metrical ones, in the following analysis. Multiple imputations were performed to fill in the missing values. For each of the 100-imputed datasets, all independent variables were combined in a Cox regression model and a backward variable selection was applied. The variables that remained after the variable elimination over all datasets more than 95 times were included in the final multivariable Cox regression model for multiple imputed datasets. Because of the known dependency between both mast cell markers (MCT and CD117), multiple Cox regression analysis was performed twice, separately for each marker. The incidence of first local recurrence was investigated by competing risk regression to consider death as a competing event. The same procedure as described above was applied to obtain the final competing risk regression model. Group comparisons were conducted by t-test, Wilcoxon rank-sum or signed-rank test, and Fisher’s exact test. The significance level was set to alpha = 5% for all statistical tests. All analyses were performed with the statistical software R (version 3.4.0, www.r-project.org) using the R package ‘cmprsk’ for the competing risk regression and the package ‘mice’ for the imputation of the missing values.

### Data availability

All data included in the present study is available over the corresponding author.

## Results

### Histological evaluation

In all tissue samples, MCT^+^ and CD117^+^ mast cells could be observed, which were mainly located in the tumour-associated stroma (Figs 1A and 2A). MCT^+^ mast cell density ranged from 0.23 cells/mm^2^ to 238.3 cells/mm^2^ [mean 38.24 cells/mm^2^+/−32.59 standard deviation (SD)]. CD117^+^ mast cell density ranged from 0.63 cells/mm^2^ to 208.29 cells/mm^2^ [mean 34.46 cells/mm^2^+/−29.95 SD]. The signed-rank test revealed a significantly higher MCT^+^ mast cell density compared to the CD117^+^ mast cell density (p = 0.0251). No differences between the MCT^+^ and CD117^+^ mast cell density between highly- (G1) and moderately/poorly-differentiated (G2/G3) OSCC could be observed (p = 0.437 and p = 0.9107, respectively).

### Oversall survival

The 18%-quantile with a value of 14.87 mast cells/mm^2^ stroma was chosen as cut-off for MCT^+^ [p < 0.001; HR 0.38; 95% CI (0.22, 0.67)] and the 60%-quantile with a value of 33.19 mast cells/mm^2^ stroma as cut-off for CD117^+^ [p = 0.031; HR 0.55; 95% CI (0.32, 0.95)].

The multivariable analysis for MCT revealed a significant influence of the MCT^+^ mast cell density [p = 0.0027; HR 0.41; 95% CI (0.23, 0.74)], the age at primary diagnosis [p = 0.0013; HR 1.04; 95% CI (1.01, 1.06)], the T stage [p < 0.001; HR 2.49; 95% CI (1.46, 4.26)], and the N stage [p = 0.0197; HR 1.86; 95% CI (1.1, 3.12)] on overall survival.

Patients with a MCT^+^ mast cell density >18%-quantile showed a significantly longer overall survival than those having a MCT^+^ mast cell density ≤18%-quantile (Fig. 1D).

The multivariable analysis for CD117 revealed a significant influence of the CD117^+^ mast cell density [p = 0.004; HR 0.44; 95% CI (0.25, 0.77)], the age at primary diagnosis [p < 0.001; HR 1.04; 95% CI (1.02, 1.06)], the T stage [p < 0.001; HR 3.15; 95% CI (1.82, 5.45)], and the N stage [p = 0.0328; HR 1.77; 95% CI (1.05, 2.98)] on overall survival.

Patients with a CD117^+^ mast cell density >60%-quantile showed a significantly longer overall survival than those with a CD117^+^ mast cell density ≤60%-quantile (Fig. 2D).

### Incidence of first local recurrence

No significant effect of the MCT^+^ and CD117^+^ mast cell density on the incidence of first local recurrence could be observed.

There was however a significant influence of the resection status (R) on the incidence of first local recurrence [p = 0.0023; HR 4.3; 95% CI (1.68, 10.99)] in the multivariable analysis.

### Correlation between the MCT^+^/CD117^+^ mast cell density and clinicopathological parameters

The analysis revealed a significant influence between the MCT^+^ mast cell density and the body weight (p = 0.0432). Patients having a MCT^+^ mast cell density ≤18%-quantile had a significantly lower body weight (mean 63.9 kg+/−12.3 kg SD) than those with a MCT^+^ mast cell density >18%-quantile (mean 70.32 kg+/− 14.22 kg SD). All other tests revealed no significant correlation between the MCT^+^/CD117^+^ mast cell density and all other clinicopathological parameters (all p-values >0.05).

## Discussion

The effects of mast cells on tumour development and progression have been investigated in different types of tumours^3–10,14–18^. In most studies, a high mast cell density was found in the surrounding tumour stroma and a tumour-promoting effect has been described^3–10^. In contrast, mast cells localized within the tumour cell clusters seem to have a tumour-suppressing effect^14–18^.

Data on the mast cell function in OSCC are rare, and conflicting observations have been described^21–24^. Lamaroon and colleagues have investigated the correlation between mast cell density and angiogenesis in OSCC immunohistochemically; they have concluded that mast cells may upregulate tumour angiogenesis via the mast cell specific tryptase^19^. Rojas *et al*. have described that mast cells may contribute to the progression of lip cancer via stimulation of angiogenesis and degradation of the extracellular matrix^22^. Ishikawa and colleagues have found that mast cell density is associated with poor prognosis and correlated with the IL-33 expression in squamous cell carcinoma of the tongue^23^. Attramadal *et al*. have quantified the mast cell density at the invasive front in tissue samples of 62 patients with T1/2 N0 M0 OSCC; they have analysed the relevance for disease recurrence. The authors have found that decreasing mast cell density with a cut-off value of 15 mast cells at the invasive front correlated with a poor prognosis^22^.

However, the comparison of the cited studies is critical because no general standards for the detection and measurement of the mast cell density have been established. Even in case of high magnification, the field of vision at the tumour-host interface often contains variable proportions of tumour and adjacent stroma. Mast cells mainly invade tumour stroma. Only a very small proportion was found in the periphery of tumour cell clusters. Therefore, this should be considered for the measurement of the mast cell density within the stroma. The quantification of mast cell hot spots at very high magnification in one field of vision predisposes selection errors. To overcome such problems, we analysed large areas at 400x magnification up to a total area of 92.6 mm^2^ and normalized the mast cell density by considering the proportion of tumour and associated stroma. Furthermore, the published studies did not compare the mast cell density with established prognostic parameters in a multivariable approach. The recent study considers the already mentioned technical aspects and compares the mast cell density with a comprehensive spectrum of established clinicopathological parameters in a large cohort of patients suffering from OSCC, as previously described^25^. High mast cell density acted as an independent marker for a favourable overall survival rate. Patients with a low density of MCT^+^ and CD117^+^ mast cells had a poorer overall survival rate compared to those with a high mast cell density.

In contrast to the transmembrane protein CD117^26^, tryptase is a mast cell-specific enzyme stored in intracellular vesicles^27^ and thus cannot be detected within mast cells after complete degranulation. Comparing MCT^+^ and CD117^+^ cell counts within the stroma; mast cells are only partially degranulated in OSCC. The number of MCT^+^ mast cells is higher than CD117^+^ mast cells. This indicates that MCT is the more sensitive marker even for partially degranulated mast cells. A combined evaluation of at least two mast cell markers should be performed to detect relative amounts of partially degranulated and non degranulated mast cells.

Shikotra *et al*. proposed a semiquantitative degranulation index score to assess the degree of mast cell degranulation^20^. This method provides additional information regarding mast cell function. However, this was not in the main focus of the recent investigation.

In contrast to Cheema *et al*.^28^, we could not find a positive association between a high mast cell density and a high grade of differentiation in OSCC by using a degranulation-sensitive and a degranulation-insensitive marker.

However, it has not been clarified why mast cells accumulate in the stroma of OSCC. The chemokine spectrum of OSCC is yet to be evaluated regarding its mast cell recruitment. In this study, we have found that an increasing mast cell density was associated with increasing body weight, which is an indicator of proper nutrition and an effective immune system. Mast cell degranulation requires an adequate stimulus^29^. If this stimulus is present and no degranulation can be observed, a potent antagonist may inhibit this stimulus^29^. We neither know the adequate stimuli in OSCC nor do we have any knowledge of specific inhibitors.

In conclusion, a high mast cell density in the tumour-associated stroma of oral squamous cell carcinoma indicates a favourable overall survival rate. We assume that this effect can be attributed to the lack of mast cell degranulation in the tumour stroma. It is, however, still unclear how mast cells suppress OSCC progression.
